# Does peak bone mass correlate with peak bone strength? Cross-sectional normative dual energy X-ray absorptiometry data in 1052 men aged 18–28 years

**DOI:** 10.1186/s12891-019-2785-8

**Published:** 2019-09-04

**Authors:** Erik Lindgren, Björn E. Rosengren, Magnus K. Karlsson

**Affiliations:** Clinical and Molecular Osteoporosis Research Unit, Department of Orthopaedics and Clinical Sciences, Lund University, Skane University Hospital, SE-205 02 Malmo, Sweden

**Keywords:** Bone, DXA, Mass, Men, Mineral, Normative, Size, Strength

## Abstract

**Background:**

Areal bone mineral density (aBMD) estimated by dual-energy X-ray absorptiometry (DXA) is used to estimate peak bone mass, define osteoporosis and predict fracture. However, as aBMD is calculated as bone mineral content (BMC) divided by the scanned area, aBMD displays an inverse relationship with bone size. In a skeleton that is increasing in size, this is a problem, as bone size is an independent factor that determines bone strength. It could therefore be questioned whether peak aBMD is the period with greatest bone strength, a period that in the hip then would occur in ages 16–19. The aim of this study was to evaluate whether there are changes in bone size in men after age 18 that may influence peak bone strength. Another aim was to provide updated normative DXA data.

**Methods:**

We scanned left femoral neck by DXA in a cross-sectional study with a population-based selection of 1052 men aged 18–28, and then registered bone mineral content (BMC, gram), aBMD (gram/cm^2^) and bone area (cm^2^) in each one-year age group. We performed analyses of variance (ANOVA) to evaluate whether there were differences in these traits between the age groups. We then used Pearson’s correlation analyses to test for trends with ageing after peak bone mass was reached.

**Results:**

We found the highest absolute femoral neck aBMD at age 19, with statistically significant differences between the one-year age groups in BMC, aBMD, and bone area (all *p* < 0.05). From peak bone mass onwards (*n* = 962), there are negative correlations between age and BMC (*r* = − 0.07; *p* < 0.05) and age and aBMD (*r* = − 0.12; *p* < 0.001), and positive correlation between age and bone area (*r* = 0.06; *p* < 0.05).

**Conclusion:**

As femoral neck bone size in young adult men becomes larger after peak bone mass, it could be questioned whether DXA estimated peak aBMD correlates with peak bone strength. We infer that aBMD must be interpreted with care in individuals with a growing skeleton, since skeletal strength may then increase, in spite of decreasing aBMD. This should be taken into account when performing DXA measurements in these ages.

## Background

The gold standard when estimating “bone mass” is areal bone mineral density (aBMD; gram/cm^2^), determined by dual-energy X-ray absorptiometry (DXA) [1–6]. As aBMD correlates with the ability of the skeleton to withstand outer forces [1, 2], the focus has been on reducing the age-related loss of aBMD, in order to decrease fragility fracture risk [1, 2]. Observational studies have further shown that one standard deviation (SD) higher aBMD is associated with halved fracture risk [3]. aBMD is therefore used in the clinical situation to identify patients with high fracture risk [4] and, applying the World Health Organization (WHO) definition, to define osteoporosis [5, 6].

However, as DXA is based on a two-dimensional imaging technique [1, 2], with aBMD derived by dividing the amount of bone mineral (bone mineral content (BMC in grams) by the scanned bone area (cm^2^), there will rise problems when estimating bone strength in growing skeletons. This is because decreased aBMD could be due to (i) decreased amount of bone minerals within an anatomic region with unchanged size, (ii) increased bone size in a region with unchanged amount of bone minerals or (iii) a combination. Reduced BMC will lead to weaker skeleton, while increased bone size, according to mechanical calculation, would increase bending strength in a long bone by the fourth power or the distance from the neutral axis. A decreased aBMD, based selectively on an increased bone size, may therefore erroneously lead to the conclusion that the individual is developing weaker bone.

High peak bone mass (PBM), the highest level of bone mass found during life [7], is important for fracture risk [8, 9] and hypothetical calculations have estimated that a 10% increase in peak aBMD could postpone the development of osteoporosis by 13 years [10]. The literature infers that hip PBM hip occurs in ages 16-19 years [11, 12]. Why the ability to withstand outer forces should start to decline at this early age seems, from an evolutionary perspective, to be counterintuitive. We therefore designed a population based cross-sectional study that included men aged 18-28 years, with the primary aim of determining whether there are changes in BMD, BMC and/or bone size from peak bone mass onwards that could influence the aBMD estimate, and if so, theoretically discussing whether PBM is the period with the greatest skeletal strength [13]. As the fracture incidence in these ages has increased during the last decade, possibly due to secular changes in bone mass [14], our secondary aim was to provide updated normative DXA-data

## Methods

**Fig. 1:**
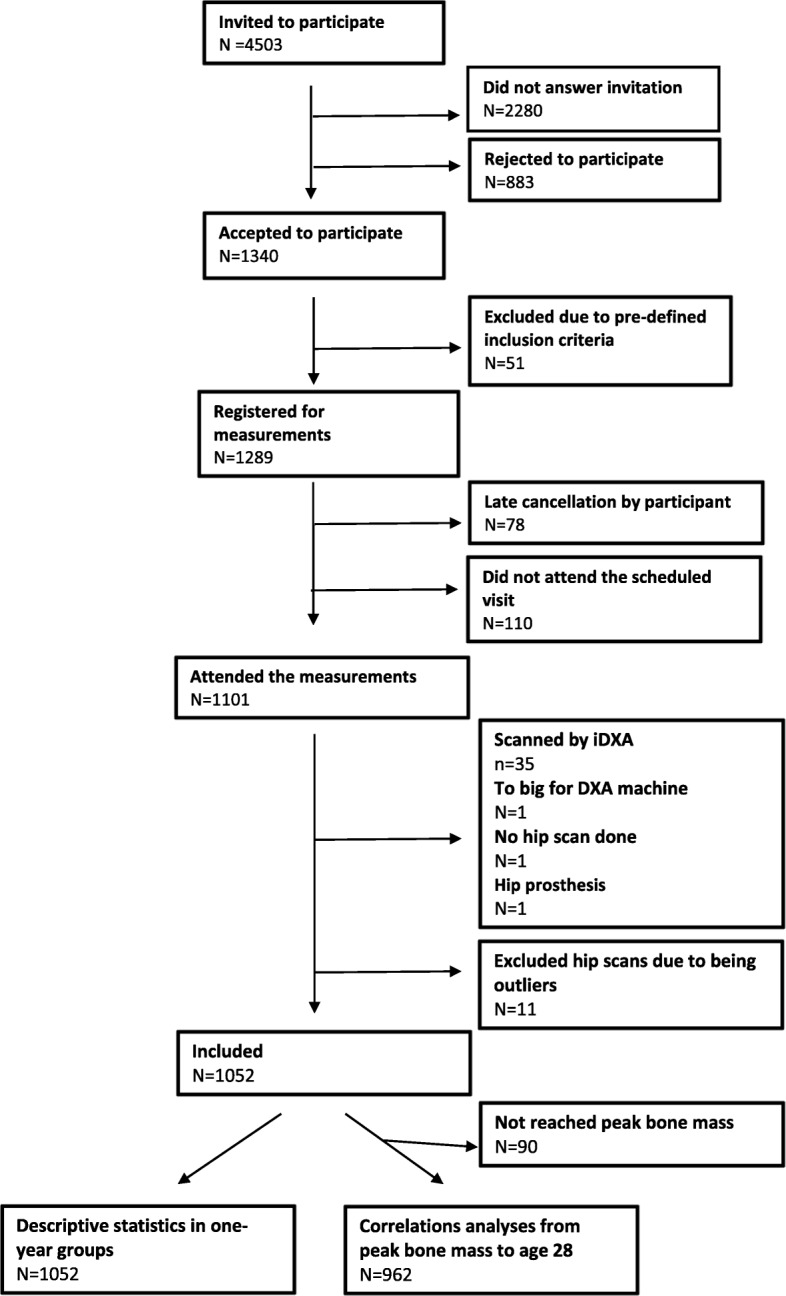
Flow-chart study population when including men aged 18–28 years

From the national Swedish official population registry, we randomly invited 4503 males aged 18 to 28 years residing in the greater city of Malmö, Sweden (population 318,107 in year 2014) by a 1-year age-stratified sampling procedure. Of these men, 2223 responded to the invitation and 1340 (60%) agreed to participate. After exclusion due to pre-specified reasons (not understanding Swedish (*n* = 1), restricted ability to move (*n* = 1), late response so that the subject was above age 28 (*n* = 26) or that the age group sample size was already completed (*n* = 23)) we had 1289 subjects who could be scanned. Then 78 individuals cancelled their participation before their scheduled visit and 110 subjects did not attend the scheduled scan without giving any explanation. The measured cohort comprised 1101 individuals, of whom 98% (*n* = 1074) were of Caucasian ethnicity (Fig. 1).

We used standard equipment to measure height in centimetres (cm), weight in kilograms (kg). Body mass index (BMI; kg/m^2^) was calculated as weight in kilograms divided by height in metres squared. We used a Lunar Prodigy scanner (GE Medical Systems, Madison, WI, USA; software version 9.20.122–9.30.044) to estimate BMC, bone area, and aBMD by left hip scans, including the regions femoral neck (FN), trochanter (Troch), and total hip (TH). We chose these regions of interest (ROI) due to the known difference in proportions of trabecular and cortical bone where FN has a pronounced cortical rim, Troch mainly trabecular bone with only a thin cortex, and TH consisting of both. By use of a total body scan we also measured total body fat mass (kg), proportion of total body fat (%) and total body lean mass (kg). Three scan technicians conducted the measurements, the scanner was calibrated daily during the study period using an inbuilt quality assessment, and three times per week using an anthropomorphic spine phantom. The short-term precision (coefficient of variation, CV) for FN, determined from duplicate scans of 14 adult subjects, was aBMD 1.6%, BMC 1.6% and bone size 1.7%.

**Fig. 2:**
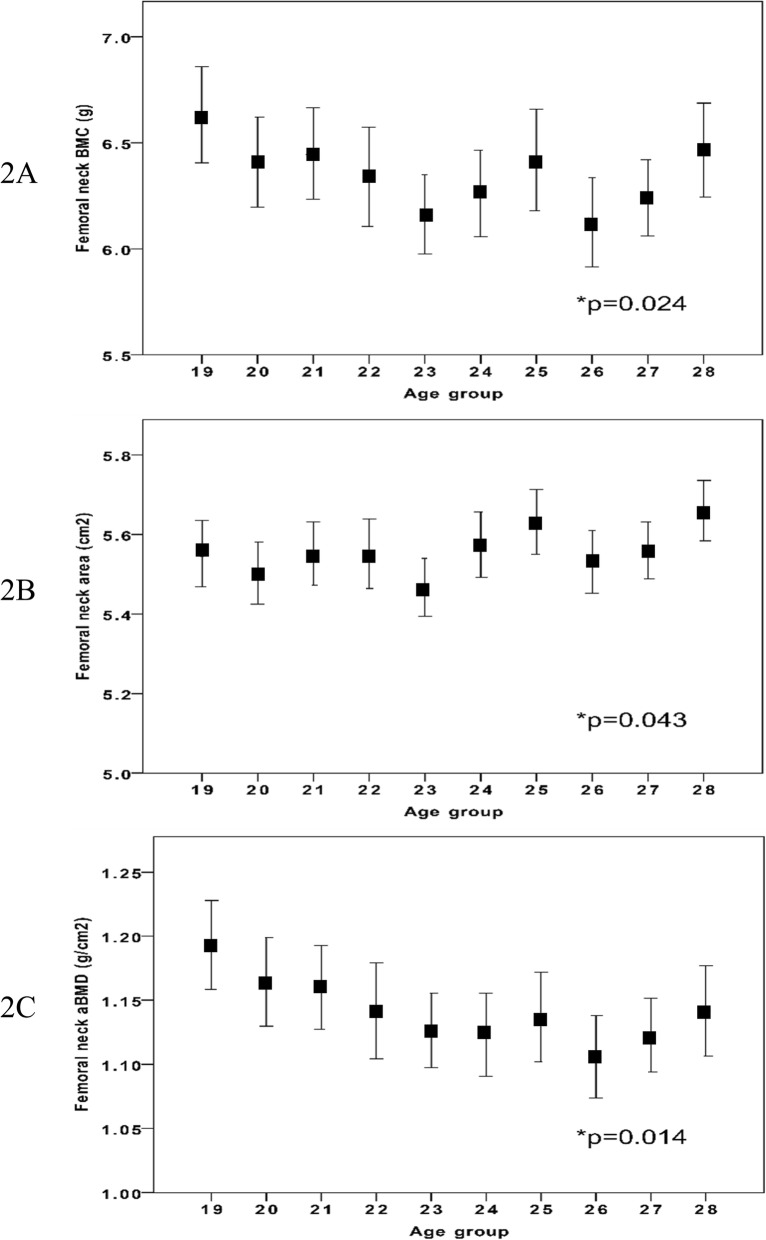
Femoral neck BMC (2**a**), area (2**b**) and aBMD (2**c**) in different age groups in 962 Swedish males aged 19 to 28 years (from peak bone mass and onwards). Age group 19 include individuals between 19.0 to 19.9 years of age etc. Data are presented as means with 95% confidence intervals. P-value represent age group differences

**Fig. 3:**
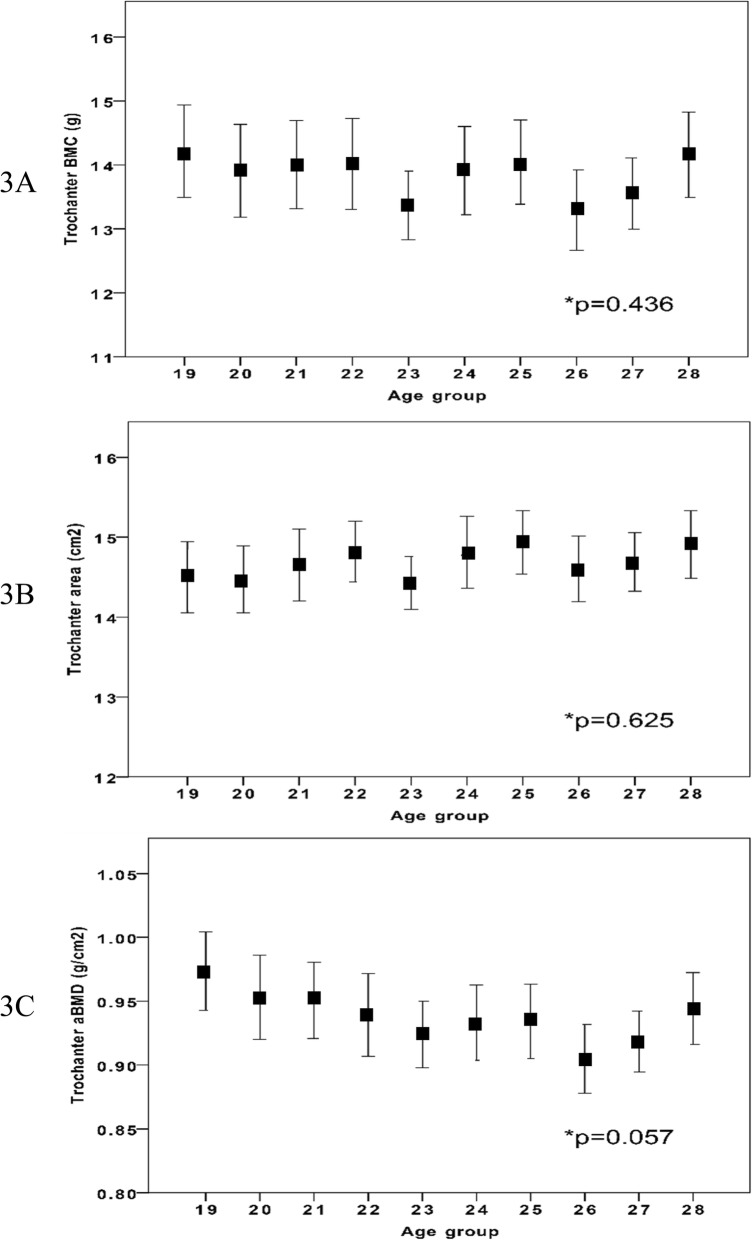
Trochanter BMC (3**a**), area (3**b**) and aBMD (3**c**) in different age groups in 962 Swedish males aged 19 to 28 years (from peak bone mass and onwards). Age group 19 include individuals between 19.0 to 19.9 years of age etc. Data are presented as means with 95% confidence intervals. *P*-value represent age group differences

**Fig. 4:**
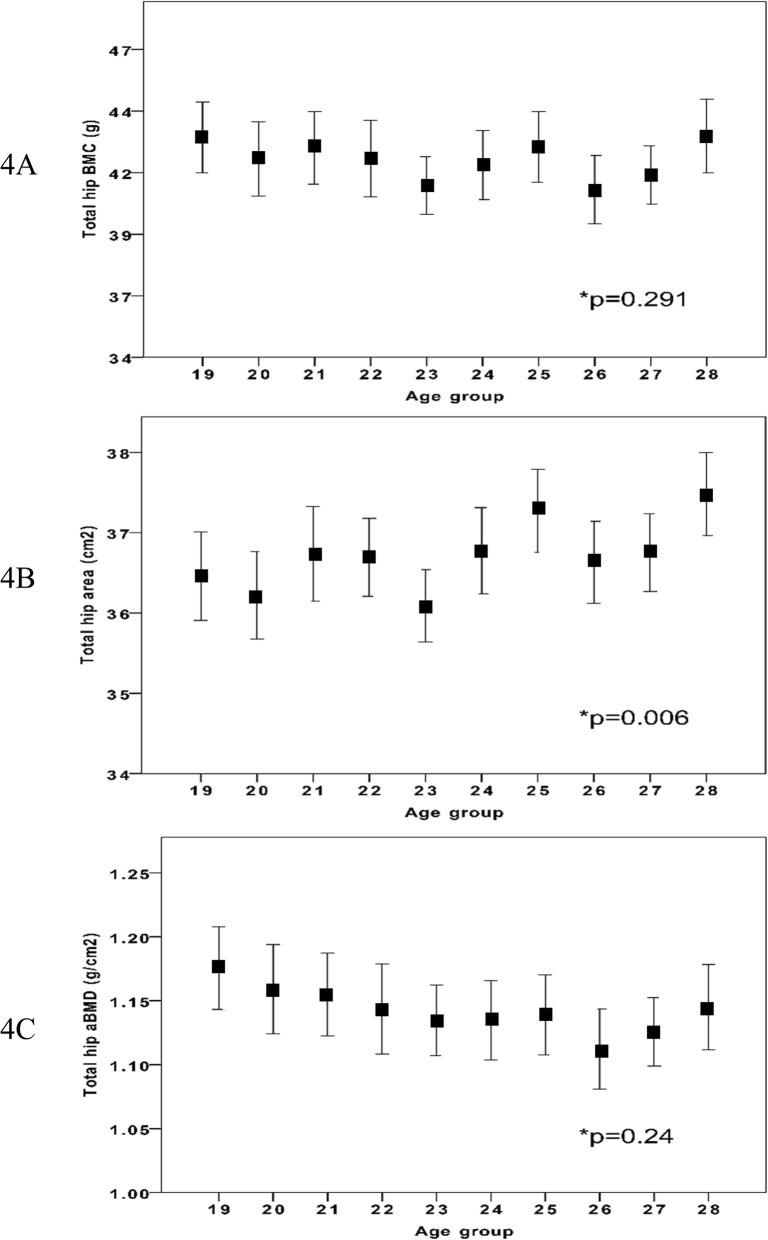
Total hip BMC (4**a**), area (4**b**) and aBMD (4**c**) in different age groups in 962 Swedish males aged 19 to 28 years (from peak bone mass and onwards). Age group 19 include individuals between 19.0 to 19.9 years of age etc. Data are presented as means with 95% confidence intervals. P-value represent age group differences

IBM SPSS Statistics for Windows (version 22.0, IBM Corp., Armonk, NY, USA) was used for all statistical analyses. Before any analysis was done, we identified and excluded for the hip scan measurements outliers (*n* = 11) by the outlier labelling rule described by Hoaglin et al. (g-value of 2.2) (Fig. 1) [16]. DXA traits are reported in 1-year age classes as means with standard deviation (SD). In graphs DXA trait values are presented in relation to increasing age as means with 95% confidence intervals (CI). Our aim was then to evaluate whether there were any differences between the 11 specific age groups, and if so, whether there was a trend from peak bone mass an onwards with ageing. Our aim was thus not to evaluate differences between two different age groups. For this reason, we used analysis of variance (ANOVA) to examine differences between the groups (*n* = 1052) and Pearson’s correlation analysis to examine correlations between the traits from PBM with increasing age as a continuous variable (*n* = 962) (Figs. 2, 3 and 4). We considered a *p*-value of less than 0.05 as a statistically significant difference.

## Results

Among the 1101 measured participants, we excluded 35 participants who were scanned with a different DXA machine (iDXA) [15], 1 who exceeded the maximal body size of the DXA-apparatus, 1 with a metallic left hip implant and 1 who did not have a hip scan conducted. Together with the excluded hip scan outliers (see statistics section, *n* = 11), we therefore achieved 1052 participants with usable Lunar Prodogy hip scans for this report (Fig. 1).

Anthropometry and left hip BMC, bone area, and aBMD are presented in Table 1. There were statistically significant age group differences in FN for BMC, bone area and aBMD (all *p* < 0.05) (Fig. 2). For the Troch bone traits, we found no statistically significant age group differences. For TH, we found statistically significant age group differences in bone area (*p* = 0.006) but not in BMC or aBMD..

**Table 1:** Normative anthropometry and DXA-data on 1052 young Swedish males aged 18–28 years

Age-group	18.00–18.99	19.00–19.99	20.00–20.99	21.00–21.99	22.00–22.99	23.00–23.99
Mean age (years)	18.6	19.3	20.4	21.5	22.4	23.4
Numbers	90	97	93	98	91	106
Weight (kg)	75.1 (13.2)	76.6 (12.1)	78.0 (13.4)	81.1 (16.1)	79.8 (13.6)	75.5 (10.5)
Height (cm)	181.9 (7.0)	181.4 (7.5)	180.8 (6.9)	181.8 (6.5)	181.9 (6.8)	180.1 (6.4)
BMI (kg/m^2^)	22.7 (3.6)	23.2 (3.1)	23.8 (3.5)	24.5 (4.4)	24.2 (4.1)	23.3 (3.1)
Total body fat mass (kg)	14.3 (9.6)	14.2 (8.1)	15.9 (8.9)	16.5 (10.5)	17.3 (10.3)	15.1 (7.8)
Total body lean mass (kg)	57.8 (6.5)	59.1 (7.3)	59.0 (6.7)	60.2 (7.1)	59.1 (6.5)	57.1 (6.0)
Femoral neck						
BMC (g)	6.42 (1.08)	6.63 (1.13)	6.41 (1.02)	6.45 (1.07)	6.34 (1.12)	6.16 (0.97)
Area (cm^2^)	5.56 (0.40)	5.55 (0.41)	5.50 (0.38)	5.55 (0.40)	5.55 (0.42)	5.44 (0.38)
aBMD (g/cm^2^)	1.15 (0.16)	1.19 (0.17)	1.16 (0.17)	1.16 (0.16)	1.14 (0.18)	1.13 (0.15)
Trochanter area						
BMC (g)	13.62 (3.23)	14.22 (3.59)	13.91 (3.52)	14.01 (3.44)	14.02 (3.41)	13.37 (2.79)
Area (cm^2^)	14.41 (1.90)	14.50 (2.20)	14.47 (2.04)	14.65 (2.24)	14.82 (1.82)	14.43 (1.71)
aBMD (g/cm^2^)	0.94 (0.15)	0.97 (0.15)	0.95 (0.16)	0.95 (0.15)	0.94 (0.15)	0.92 (0.13)
Total hip						
BMC (g)	41.50 (6.90)	42.93 (7.17)	42.06 (7.30)	42.50 (7.32)	42.07 (7.44)	40.98 (6.11)
Area (cm^2^)	36.47 (2.55)	36.46 (2.74)	36.22 (2.64)	36.73 (2.94)	36.69 (2.33)	36.09 (2.34)
aBMD (g/cm^2^)	1.14 (0.16)	1.18 (0.16)	1.16 (0.17)	1.16 (0.16)	1.14 (0.17)	1.13 (0.14)
Age-group	24.00–24.99	25.00–25.99	26.00–26.99	27.00–27.99	28.00–28.99	All age groups
Mean age (years)	24.4	25.4	26.4	27.5	28.5	23.5
Numbers	84	97	98	112	86	1052
Weight (kg)	78.3 (13.3)	80.0 (10.3)	82.4 (14.5)	79.7 (12.4)	83.3 (12.2)	79.1 (13.1)
Height (cm)	181.4 (7.0)	180.7 (6.2)	181.5 (6.5)	180.7 (7.1)	182.6 (6.4)	181.3 (6.8)
BMI (kg/m^2^)	23.8 (3.7)	24.5 (2.9)	25.0 (4.6)	24.4 (3.5)	25.0 (3.4)	24.0 (3.7)
Total body fat mass (kg)	15.7 (9.1)	17.7 (8.1)	19.3 (11.3)	17.1 (8.8)	18.3 (9.1)	16.5 (9.4)
Total body lean mass (kg)	59.3 (7.0)	59.0 (6.8)	59.6 (6.7)	59.2 (6.6)	61.4 (7.1)	59.1 (6.8)
Femoral neck						
BMC (g)	6.26 (0.94)	6.42 (1.19)	6.13 (1.04)	6.24 (0.96)	6.47 (1.04)	6.35 (1.06)
Area (cm^2^)	5.57 (0.38)	5.63 (0.40)	5.53 (0.40)	5.56 (0.38)	5.66 (0.35)	5.56 (0.39)
aBMD (g/cm^2^)	1.12 (0.15)	1.14 (0.17)	1.11 (0.16)	1.12 (0.15)	1.14 (0.16)	1.14 (0.16)
Trochanter area						
BMC (g)	13.91 (3.18)	14.04 (3.26)	13.30 (3.13)	13.55 (2.98)	14.16 (3.11)	13.81 (3.24)
Area (cm^2^)	14.81 (2.08)	14.94 (1.96)	14.60 (2.05)	14.69 (1.96)	14.91 (1.97)	14.65 (2.00)
aBMD (g/cm^2^)	0.93 (0.14)	0.93 (0.14)	0.90 (0.13)	0.92 (0.13)	0.94 (0.13)	0.94 (0.14)
Total hip						
BMC (g)	41.81 (6.47)	42.55 (7.12)	40.81 (6.88)	41.40 (6.29)	42.99 (6.95)	41.94 (6.91)
Area (cm^2^)	36.78 (2.47)	37.27 (2.57)	36.63 (2.55)	36.75 (2.59)	37.48 (2.42)	36.68 (2.58)
aBMD (g/cm^2^)	1.13 (0.14)	1.14 (0.16)	1.11 (0.16)	1.13 (0.14)	1.15 (0.16)	1.14 (0.16)

*BMI* body mass index, *BMC* bone mineral content, *aBMD* areal bone mineral density

Data are presented as means with 1 standard deviation bracketed. Age group 18 contains individuals between 18.0 to 18.9 years of age etc.

Peak aBMD (the highest absolute aBMD value) was found in age group 19 (Table 1). After peak aBMD (from age 19) we found a negative correlation between age and FN BMC (r = − 0.07; *p* < 0.02) and age and FN aBMD (− 0.12, *p* < 0.001) while we for FN bone area we found a positive correlation with age (*r* = 0.06; *p* < 0.05) (Table 2). For Troch traits we found a negative correlation between age and Troch aBMD (*r* = − 0.10; *p* < 0.01) (Table 2). For TH traits we found a negative correlation between age and TH aBMD (*r* = − 0.09; *p* < 0.01) and a positive correlation between age and TH area (*r* = 0.09; *p* < 0.01).

**Table 2:** Pearson’s correlation analyses (r) between age and DXA-traits from peak bone mass (PBM) and onward in 962 Swedish males aged 19 to 28 years. Statistical significance is bolded

		Correlation coefficient (r)	*p*-value
**Femoral neck**	Age*BMC	−0.074	**0.02**
	Age*area	0.064	**0.048**
	Age*aBMD	−0.116	**<0.001**
**Trochanter area**	Age*BMC	−0.038	0.24
	Age*area	0.045	0.16
	Age*aBMD	−0.095	**<0.01**
**Total hip**	Age*BMC	−0.032	0.32
	Age*area	0.094	**<0.01**
	Age*aBMD	−0.087	**<0.01**

*BMC* bone mineral content, *aBMD* areal bone mineral density

## Discussion

In this paper, which also present normative hip DXA data for men aged 18–28 years, we found peak hip aBMD at age 19. After peak aBMD, FN bone area became larger with older ages,, although with a weak correlation, while BMC (the total amount of bone mineral) declined. Our findings suggest that the lower FN aBMD after peak bone mass, seems to be due not only on to a decline in BMC, but also a gain in bone size. Since bone size is an independent factor that determines bone strength, we will hypothetically discuss below the implications that this could have when estimating skeletal strength and bone resistance to trauma.

We infer with this paper that, in expanding skeletons, aBMD ought to be interpreted with care, as the bone resistance to fracture may increase, even if repeated DXA scans show decreasing aBMD. This should be taken into account, not the least when repeated DXA measurements are conducted in these ages. Furthermore, we found an increase in bone size until age 28, that is, this study cannot suggest if or when the age related expansion in periosteal width ceases. In fact, the periosteal expansion could possibly be lifelong, as there are data in women are data that support an age-related periosteal expansion (in conjunction with a medullary expansion) with ageing from menopause until age 71, simultaneous with a decrease in aBMD [17]. In cited study, the increase in skeletal width was not associated with growth and modelling, but instead with decreasing oestrogen level and remodelling [17].

The reduction in aBMD that follows the decline in BMC and increase in bone size, is contradictory in respect to the bone resistance to fracture, since both traits are independently associated with fracture risk [18] and the structural strength of the bone [19]. In fact, bone size may be of more importance for bone strength than the amount of minerals, as the resistance to bending of a tubular structure is proportional to the fourth power of the distance from the neutral axis [20]. In summary, the lower aBMD found in this report could therefore erroneously be interpreted to suggest that the hip becomes more fragile after age 19, while it in fact may be the opposite.

Our results show low coefficients of correlation between age and the respective DXA traits. Low correlation coefficients, and subsequently low coefficients of determination (r^2^), may be due to the great natural variance in our selected bone traits, where to age additional factors besides age, such as height, weight, genetics, physical activity level and dietary calcium intake level, could influence the outcome. It must also be taken into consideration that our results may be influenced by secular changes in Sweden during the 5 years of data collection (year 2006 to 2011). However, we believe that any such changes would be minimal. This view is supported by the annual report by the National Swedish Board of Health and Welfare, and the National Swedish Public Health Institute that between 2004 and 2011 found no changes in Swedish men in sedentary time, only a minimal decrease in smoking habits, and a stable prevalence of obesity [21].

Ever since DXA-derived aBMD was included when defining osteoporosis [5, 6], researchers have focused on aBMD when estimating bone strength. Low aBMD has also been shown to predict fractures [22–25]. However, it is important to realize that aBMD only reflects part of the traits that contribute to bone strength. aBMD is a derived from BMC divided by the two-dimensional scanned area and not the volume, thus leading to an overestimation of volumetric BMD (“true BMD”) in larger bones and an underestimation in smaller bone [19, 26]. The complex term aBMD when describing a three-dimensional structure should therefore only be considered as a surrogate measure for bone strength. We must instead accept that DXA cannot determine the true three-dimensional structure and only part of the ability to withstand external forces. One reason for this is partly the non-uniform cross-sectional distribution of cortical and trabecular bone around the central axis. This bias may be dealt with by using standardized scanning methods, making the angle of perspective similar in all scans. But there is more structural change that may affect bone resistance to trauma, as intra-cortical porosity, being greater in old than young individuals, not captured by DXA. Other properties that DXA cannot identify are abnormalities in bone microarchitecture and bone turnover, factors that also contribute to bone strength.

Study strengths include the large sample in each age group and the few and highly experienced technicians conducting the scans. We underline that the data are cross-sectional, which makes estimation of individual changes impossible. When interpreting our data, the sensitivity of the DXA method must be taken into account. Usually, it is recommended that repeated DXA measurement should be considered with a minimum 2–3 years apart. However, this applies when evaluating the aBMD value. We emphasize that DXA is not a method primarily to determine skeletal structure and skeletal width, suggesting that small changes in skeletal width may not be detected within a period of 2–3 years between two measurements. During the evaluated years there are also changes in soft tissue composition (Table 1), as well as medullary fat content, changes that could interfere with the aBMD estimate, then leading to erroneous conclusions about changes in bone mass. Another study limitation is the inclusion of only males. Future research is needed and would benefit from inclusion of prospective data on both sexes and scanning of different parts of the skeleton, also in older ages, as to evaluate whether bone area in hip continues to increase. Finally, this study could only discuss the amount of bone mineral and bone size in relation to skeletal strength in a hypothetical perspective, since no mechanical tests were included. Future studies should therefore prospectively follow men and women into older ages to see whether the increase in bone size continues and include more accurate measuring techniques (such as high-resolution peripheral computed tomography; pQCT). This method could then evaluate other structural parameters of importance for bone strength. Finally, future studies should also include mechanical breaking tests, to verify or refute the hypothetical inferences as regards bone strength put forward in this study. By doing this, we would arrive at a better understanding of the development of the human skeleton throughout life.

## Conclusion

Since the hip in young adult men increases in size during the third decade in life, resulting in a decline in aBMD (the amount of minerals divided by bone area), we question whether peak bone aBMD correlates with peak bone strength. We infer that aBMD should not be used uncritically in a growing skeleton to define peak bone strength.

## Data Availability

The datasets generated and/or analysed during the current study are not publicly available. The reason is that the ethical ethical review board does not allow us to send data abroad or publish individualized data. Data and analysed data are available through the corresponding author on reasonable request.

## Abbreviations

aBMD: areal bone mineral density
ANOVA: analyses of variance
BMC: bone mineral content
BMI: body mass index
cm: centimetres
CV: coefficient of variation
DXA: Dual-energy X-ray absorptiometry
FN: femoral neck
kg: kilograms
PBM: peak bone mass
pQCT: peripheral computed tomography
ROI: regions of interest
SD: standard deviation
TH: total hip
Troch: trochanter
WHO: World Health Organization
